# Effect of Underlying Cardiovascular Disease on the Prognosis of COVID-19 Patients; a Sex and Age-Dependent Analysis

**DOI:** 10.22037/aaem.v9i1.1363

**Published:** 2021-09-30

**Authors:** Mohammad Haji Aghajani, Ziba Asadpoordezaki, Mehrdad Haghighi, Asma Pourhoseingoli, Niloufar Taherpour, Amirmohammad Toloui, Mohammad Sistanizad

**Affiliations:** 1 Prevention of Cardiovascular Disease Research Center, Shahid Beheshti University of Medical Sciences, Tehran, Iran.; 2 Department of Psychology, Maynooth University, Kildare, Ireland.; 3 Kathleen Lonsdale Institute for Human Health Research, Maynooth University, Kildare, Ireland.; 4 Infectious Diseases and Tropical Medicine Research Center, Shahid Beheshti University of Medical Sciences, Tehran, Iran.; 5 Physiology Research Center, Iran University of Medical Sciences, School of Medicine, Tehran, Iran.; 6 Department of Clinical Pharmacy, School of Pharmacy, Shahid Beheshti University of Medical Sciences, Tehran, Iran.

**Keywords:** COVID-19, Hypertension, Coronary Artery Disease, Prognosis

## Abstract

**Introduction:**

Adults with underlying medical disorders are at increased risk for severe illness from the virus that causes COVID-19. This study aimed to compare the effect of underlying diseases on the mortality of male and female patients as a primary objective. We also evaluated the effect of drugs previously used by COVID-19 patients on their outcome.

**Methods:**

This retrospective cohort study was carried out on confirmed cases of COVID-19 who were admitted to a teaching hospital in Tehran, Iran. Data was gathered from patients’ files. Log binomial model was used for investigating the association of underlying diseases and in-hospital mortality of these patients.

**Results:**

A total of 991 patients (mean age 61.62±17.02; 54.9% male) were recruited. Hypertension (41.1%), diabetes mellitus (30.6%), and coronary artery disease (19.6%) were the most common underlying diseases. The multivariable model showed that hypertension (RR = 1.62; 95% CI: 1.22-2.14, p = 0.001) in male patients over 55 years old and coronary artery disease (RR = 2.40; 95% CI: 1.24-4.46, p = 0.009) in female patients under 65 years old were risk factors of mortality. In females over 65 years old, the history of taking Angiotensin Converting Enzyme inhibitors (ACEi) and Angiotensin Receptor Blockers (ARB) (RR = 0.272; 95% CI: 0.17-0.41, p = 0.001) was a significant protective factor for death.

**Conclusions:**

COVID-19 patients with a history of cardiovascular diseases such as hypertension and coronary artery disease, especially those in specific age and sex groups, are high-risk patients for in-hospital mortality. Additionally, a previous history of taking ACEi and ARB medications in females over 65 tears old was a protective factor against in-hospital mortality of COVID-19 patients.

## 1. Introduction

Coronaviruses are a large family of viruses that are known to cause illnesses ranging from the common cold to more severe diseases such as Middle East Respiratory Syndrome (MERS) and Severe Acute Respiratory Syndrome (SARS)(1, 2). The severe acute respiratory syndrome coronavirus 2 (SARS-CoV-2), which causes COVID-19 was first reported in Wuhan, China, in late December 2019 (3). COVID-19 has spread worldwide leading to a global pandemic, it affects different areas of human life such as health, social, and economy, and had caused 93,805,612 confirmed cases and 2,026,093 deaths, by 18 January 2021 (4).

Adults with underlying medical disorders are at increased risk for severe illness from the virus that causes COVID-19. Cardiovascular disease (CVD) is one of the most important underlying diseases, which could affect the prognosis of patients with COVID-19 (5). In addition, a high rate of underlying CVD has been observed in patients with COVID-19, and increased mortality rates have been reported with these comorbidities (6, 7). 

From the point of view of studies from different countries, age and sex are considered to be strong prognostic factors of death in patients with COVID-19. Sex difference in COVID-19 outcome results from an interlock interaction between biological, geographical, and social impacts, and past medical history including preexisting CVD. 

This study aimed to compare the effect of underlying disease on the mortality of male and female patients as a primary objective. We also evaluated the effect of drugs previously used by COVID-19 patients, including Beta blockers, Angiotensin Converting Enzyme inhibitors (ACEi), Angiotensin Receptor Blockers (ARB), anticoagulants, and antiplatelet drugs, on their outcome. 

## 2. Methods

***2.1. Study Design and Setting***

The present study was a retrospective cohort study conducted on 991 confirmed COVID-19 patients with hospitalization criteria in Imam Hossein Hospital, affiliated to Shahid Beheshti University of Medical Sciences, Tehran, Iran. The current study was performed based on Helsinki declarations and was approved by the reviewer’s board and ethics committee of the deputy for research affairs, Shahid Beheshti University of Medical Sciences, Tehran, Iran (Ethics code: IR.SBMU.RETECH.REC.1399.263).

***2.2. Participants***

Using the census method, all of the patients who were admitted from 29 February to 20 July 2020 with a laboratory confirmed SARS-Cov2 infection based on Reverse Transcriptase Polymerase Chain Reaction test (RT-PCR) using throat and nose swab specimens were included in this study. Confirmed COVID-19 outpatients and the patients with the clinical diagnosis of COVID-19 whose diagnosis was not confirmed by PCR test were excluded from the study. 

***2.3. Follow-up and outcome***

In this study, the measured outcome was in-hospital mortality and follow-up time was the duration of hospitalization, which is from the date of admission to date of discharge or when the patient died during hospitalization. 

***2.4. Data gathering***

Data were collected from medical records of COVID-19 patients using a researcher-made checklist. Researchers designed a checklist based on the aim of the study according to the opinion of medical and methodological expert team. Data extracted for each patient included demographic characteristics (age, sex), Body Mass Index (BMI), past medical histories such as underlying diseases and medication history, signs and symptoms on admission, duration of hospitalization, and outcome of patients such as intensive care unit (ICU) admission, and in-hospital mortality. The mentioned information was extracted from medical records of COVID-19 patients by a trained research team that included nursing and medical personnel of cardiac care unit (CCU) and ICU. 

***2.5. Definitions***

In this study, underlying diseases were defined as chronic health conditions that patients already had before their hospitalization due to COVID-19 infection. Candidate underlying diseases were Diabetes Mellitus (DM), Central Nervous System (CNS) disorders, Hypertension (HTN), Chronic Kidney Diseases (CKDs), any type of cancer, Hyperlipidemia, Immunosuppressive disorders, Respiratory diseases, Congenital disorders, Coronary Artery Diseases (CADs), and history of coronary angioplasty or Coronary Artery Bypass Graft (CABG) surgery. The information from the mentioned diseases was based on the reports of physicians’ examination.

Medication history referred to the patients’ use of different types of drugs due to their special health conditions, based on a physicians’ prescription, before hospitalization due to COVID-19 infection. In this study, the studied medications were Beta blockers, ACEi or ARB, ASA (Acetyl Salicylic Acid), Atorvastatin, Nitroglycerin, Warfarin, Rivaroxaban, and Metformin. 

***2.6. Statistical Analysis***

Continuous variables were described using mean ± standard deviation (SD), and categorical variables were expressed as frequency (percentage). The normality assumption was examined using checking kurtosis, skewness, box plot, and Q-Q plot, due to the large amount of data. T-test and Mann–Whitney U test were used for comparisons of means in normal and non-normal variables, respectively. In addition, a multivariable log binomial regression model was performed for investigating the association of in-hospital mortality with underlying diseases and other variables of the study. The final multivariable model was selected based on potential risk factors according to the backward approach with P-value < 0.2. Due to the proven role of sex and age in the etiology of disease and its prognosis, we used subgroup analysis to consider the probable effect of these interactive biological variables. In subgroup analysis, the age cut-offs considered for males and females were 55 and 65 years, respectively (8). Findings were reported as Relative Risk (RR) and 95% confidence interval (95% CI). A two-sided P-value less than 0.05 was considered statistically significant. Analyzing was done using the STATA 14 Package.

## 3. Results

***3.1. Demographic characteristics*** ***and clinical findings***

Among the 991 patients, 544 (54.9%) were male and 257 patients (25.9%) died. The mean age was 61.62±17.02 years [range 10-99]. The most common chief complaints were dyspnea with 626 (63.2%), cough with 524 (52.9%), fever with 495 (49.9%), myalgia with 320 (32.3%), nausea/vomiting with 204 (20.6%), and diarrhea with 90 (9.1%) cases. The median duration of hospitalization was 6 days with an inter quartile range (IQR) of 6. One hundred eighteen (11.9%) patients were admitted to ICU. Table 1 shows the distribution of demographic and some clinical characteristics of studied cases.

***3.2. Underlying diseases and past cardiovascular medications***

Hypertension with 407(41.1%), Diabetes Mellitus with 303(30.6%), and CAD with 194(19.6%) cases were the most frequent underlying diseases in both sexes. In the first step of investigating the association of the underlying diseases with death in our whole population of COVID-19 patients, the univariate analysis showed that CNS disorders (17.2% vs 8.6% in dead and alive patients, respectively with p <0.001), HTN (53.7% vs 36.6% dead and alive patients, respectively with p <0.001), and CAD (25.7% vs 17.4% dead and alive patients, respectively with p <0.001) were underlying diseases associated with death. Also, having a history of using ASA (25.2% vs 18.7% dead and alive patients, respectively with p = 0.026), nitroglycerin (10.5% vs 6.3% dead and alive patients, respectively with p = 0.027), and Warfarin or Rivaroxaban (7% with 2.9% dead and alive patients, respectively with p = 0.003) had a significant association with mortality in our whole population. Tables 2 and 3 show the association between underlying diseases and history of using cardiovascular medications with mortality of patients based on their sex.

In the next step of designing a model, we fitted a multivariable model, adjusting the effects of demographic factors. In this model, only demographic factors of sex and age had a significant association with death. Accordingly, we have noticed the strong effect of sex and age and their interactions on this model. To adjust their interaction effects precisely and to know how underlying diseases affect mortality in each sex, we have analyzed the relation between mortality, underlying diseases and medication history in different age and sex subgroups. Our final multivariable models were fitted in four different sex and age groups. A total number of 257 Females under 65, 190 females over 65, 199 males under 55, and 345 males over 55 were our subgroups in the final model.

For males over 55 years old, HTN was a significant risk factor in both univariate and multivariable analyses with RR: 1.34 (95% CI: 1.03-1.74, P=0.029) and RR: 1.62(95% CI: 1.22-2.14, P=0.001), respectively. There was no variable that significantly associated with mortality in males under 55 years old. 

In females under 65 years old, CAD with RR: 3.01(95% CI: 1.55-5.84, P=0.001) in univariate analysis, and RR: 2.40 (95% CI: 1.24-4.46, P=0.009) in Multivariable analysis was a remarkable risk factor of death on both analyses. Although the history of taking ACEi or ARB with RR: 2.16 (95% CI: 1.18-4.03, P=0.012), and Atorvastatin with RR: 2.45(95% CI: 1.29-4.67, P=0.006) were significant risk factors in univariate analyses, they were not significant in multivariable analysis. 

In females over 65 years old, history of taking ACEi or ARB was a significant protective factor against death in both univariate and Multivariable analyses with RR: 0.521 (95% CI: 0.32-0.83, P=0.007) and RR: 0.272 (95% CI: 0.17-0.41, P=0.001), respectively. The results of univariate and Multivariable models are shown in table 4.

## 4. Discussion

In this retrospective cohort study, we investigated the association between underlying cardiovascular diseases, patients’ drug history, and COVID-19 mortality. We found that in males older than 55, HTN and in females under 65, coronary artery disease was strongly associated with in-hospital mortality. Additionally, a previous history of taking ACEi and ARB medications in females over 65 were protective factors against in-hospital mortality of COVID-19 patients.

There are several studies on the assessment of the relationship of a history of HTN and severity of COVID-19 and its related mortality. Several studies showed that HTN is a risk factor for COVID-19-related mortality. For example, a cohort study showed that Hypertensive COVID-19 patients have more severe inflammatory responses to the disease and experience more severe internal organ injury. Poor outcome in hypertensive patients was more prevalent than non-hypertensive COVID-19 patients (9). Meanwhile, other studies demonstrated that there was no adequate evidence supporting the prognostic effect of HTN for COVID-19 (10). Our results showed that HTN is a risk factor for mortality only in males older than 55 years. This finding justifies the inconsistency between the studies. Because hypertension seems to be a risk factor for patient mortality in a certain group of patients, not for all COVID-19 patients.

To be illustrated, our findings are in line with previous research, confirming the prognostic value of age in predicting COVID-19 patients’ disease severity and its pertaining mortality (11). Moreover, we have concluded that the male sex is independently associated with a higher risk of death in COVID-19 patients. Evidence regarding the impact of sex on in-hospital mortality of COVID-19 patients is a growing topic and so far, independent association of male sex with mortality has been shown in some studies (12-14). Reasons behind this finding could be the higher levels of humoral and cellular immunity in females and possible differences in sex-based comorbidities (14-19).

Underlying cardiovascular disease was a risk factor of in-hospital mortality in female patients aged less than 65 years. It has been previously shown that premenopausal females who develop coronary artery disease might have lower levels of estrogen compared to those without coronary artery disease (20-22). Besides, there is growing evidence on the protective effect of estrogen hormone against COVID-19 (23-26). Considering that the most important factor responsible for higher levels of immune system activity in females is probably hormonal differences, there seems to be a correlation between lower estrogen levels, higher incidence of CAD, more susceptibility to developing severe disease from SARS-CoV-2 infection, and higher mortality rates.

Growing evidence suggests that taking anti-hypertensive drugs (ACEI/ARB) is not associated with higher mortality rates or illness severity in COVID-19 patients and in fact, it might be beneficial for these patients. We demonstrated that the history of taking these drugs has a protective impact against the mortality of females more than 65 years old, in line with other studies showing a possibly lower mortality rate in patients treated with these medications (6, 27-30). However, the effects of taking these medications haven’t been completely studied in different ages and sexes. Due to complexity regarding confounding factors of underlying diseases and biological changes, especially in females during post-menopausal period, more studies are required to assess the effects of these drugs in specific age categories.

**Table1:** Baseline characteristics based on final disease outcome

p-value	Death ((n:257	Discharged ( (n:734	Total (n:991)	**Variables**
				**Sex (%)**
0.020*	100(38.9)	347(47.3)	447(45.1)	Female
	157(61.1)	387(52.7)	544(54.9)	Male
				**Age (Year)**
<0.001^*^	70.84±14.23	58.45±16.75	61.62±17.02	Mean ± SD
				Signs and Symptoms
0.395	168(65.4)	458(62.4)	626(63.2)	Dyspnea
0.252	128(49.8)	396(54)	524(52.9)	Cough
0.285	121(47.1)	374(51)	495(49.9)	Fever
0.001*	60(23.3)	260(35.4)	320(32.3)	Myalgia
0.635	50(19.5)	153(20.8)	203(20.5)	Nausea/ Vomiting
0.110	17(6.6)	73(9.9)	90(9.1)	Diarrhea
				**ICU Admission **
<0.001^*^	74(28.8%)	44(6%)	118(11.9%)	Yes
	183(71.2%)	690(94%)	873(88.1%)	No
				**BMI(Kg/M2)**
0.170	25.92(5.87)	26.23(5.18)	26.17(5.31)	Median (IQR)
				**Hospital Stay (Day) **
0.338	6(7)	6(5)	6(6)	Median (IQR)

Data are presented as mean ± standard deviation (SD), number (%) or median (inter quartile range). * P<0.05 was statistically significant. BMI: Body Mass Index; ICU: intensive care unit; IQR: inter quartile range.

**Table 2:** Distribution of underlying diseases and their crude association with in-hospital mortality between sex groups in COVID-19 patient

**Variables**	**Male (n=544)**			**Female** ** (n:447)**		
	**Survived**	**Dead**	**P **	**Survived**	**Dead**	**P **
**DM**	112(28.9)	50(31.8)	0.50	106(30.5)	35(35.0)	0.39
**CNS**	44(11.4)	32(20.4)	0.006*	19(5.5)	12(12.1)	0.02*
**Hypertension**	121(31.3)	76(48.4)	<0.001*	148(42.7)	62(62.0)	<0.001*
**CKD**	36(9.3)	16(10.2)	0.74	34(9.8)	16(16.0)	0.08
**Cancer **	9(2.3)	7(4.5)	0.18	20(5.8)	6(6.0)	0.92
**Hyperlipidemia**	19(4.9)	12(7.6)	0.213	21(6.1)	10(10.0)	0.17
**ISD**	9(2.3)	7(4.5)	0.18	16(4.6)	5(5.0)	0.87
**RD**	27(7.0)	16(10.2)	0.20	31(8.9)	13(13.0)	0.22
**CD**	5(1.3)	4(2.5)	0.29	10(2.9)	2(2.0)	0.63
**CAD**	77(19.9)	36(22.9)	0.42	51(14.7)	30(30.0)	<0.001*
**Coronary revisualization**						
CABG	31(8.0)	12(7.6)	0.82	7(2.0)	7(7.1)	0.003*
Angioplasty	24(6.2%)	12(7.6%)		11(3.2%)	8(8.2%)	

Data are presented as number (%). DM: Diabetes mellitus; CNS: Central nervous system disorders; CKD: Chronic kidney Disease; ISD: Immunosuppressive disorders; RD: Respiratory diseases; CD: Congenital Disorders; CAD: Coronary Artery Disease; CABG: Coronary Artery Bypass Graft; P<0.05 was considered statistically significant.

**Table 3:** Distribution of past cardiovascular medications and their crude association with in-hospital mortality between sex groups among COVID-19 patient

**Medications**	**Male (n=544)**			**Female** ** (n:447)**		
	**Survived**	**Dead**	**P **	**Survived**	**Dead**	**P **
**Beta Blockers **	51(13.2)	20(12.7)	0.862	57(16.4)	28(28)	0.009*
**ACEi or ARB **	82(21.2)	38(24.1)	0.473	106(30.5)	31(31)	0.931
**ASA **	79(20.5)	42(26.6)	0.119	58(16.7)	23(23)	0.151
**Atorvastatin **	54(14)	25(15.8)	0.582	66(19)	25(25)	0.191
**Nitroglycerin **	29(7.5)	13(8.2)	0.582	17(4.9)	14(14)	0.002*
**Warfarin/Rivaroxaban**	12(3.1)	8(5.1)	0.271	9(2.6)	10(10.0%)	0.003*

Data are presented as number (%). * P<0.05 was statistically significant. ACEi: Angiotensin Converting Enzyme Inhibitor; ARB: Angiotensin Receptor Blocker; ASA: Acetyl Salicylic Acid.

**Table 4:** Univariate and Multivariable analysis results for the association of underlying disease and drug history with in-hospital death in patients with COVID-19 in different age and sex subgroups

**Variables**	**Univariate**		**Multivariable**	
	**RR (95% CI)**	**P**	**RR (95% CI)**	**P**
Males over 55 years old**				
**Hypertension**				
No	1		1	-
Yes	1.34(1.03-1.74)	0.029*	1.62(1.22-2.14)	0.001*
**Taking Beta blocker**				
No	1		1	
Yes	0.77(0.51-1.14)	0.200	0.68(0.46-1.02)	0.063
**Taking** ** ACEi or ARB **				
No	1		1	
Yes	0.89(0.66-1.02)	0.446	0.74(0.54-1.01)	0.063
**Females under 65 years old**				
**Coronary Artery Disease**				
No	1		1	
Yes	3.01(1.55-5.84)	0.001*	2.40(1.24-4.64)	0.009*
**Taking Atorvastatin**				
No	1		1	
Yes	2.45(1.29-4.67)	0.006*	1.74(0.90-3.37)	0.096
**Taking ACEi or ARB**				
No	1		1	
Yes	2.16(1.18-4.03)	0.012*	1.72(0.92-3.20)	0.085
**Females over 65 years old**				-
**Hypertension**				
No	1		1	
Yes	1.10(0.72-1.70)	0.643	1.16(0.912-1.75)	0.086
**Taking ACEi** ** or ARB **				
No	1		1	
Yes	0.521(0.32-0.83)	0.007*	0.27 (0.17-0.41)	<0.001*
**Chronic kidney Disease**				
No	1		1	
Yes	1.51(0.969-2.33)	0.068	1.34(0.945-2.39)	0.067

CI: confidence interval. *P<0.05 was considered statistically significant. **All variables were excluded in the final model of the subgroup of males under 55 years old. ACEi: Angiotensin Converting Enzyme Inhibitor; ARB: Angiotensin Receptor Blocker.

## 5. Limitations

This retrospective study had its limitations. Due to its nature, tools to evaluate patients’ data documentation were not available; Some data such as previous medication history were recorded according to patients’ self-report and, therefore, were not totally reliable. Previous medical files of patients were inaccessible due to the shortage of time and supplies during the pandemic. 

## 6. Conclusion

COVID-19 patients with a history of cardiovascular diseases such as hypertension and coronary artery disease, especially those in specific age and sex groups, are high-risk patients for in-hospital mortality. Additionally, a previous history of taking ACEi and ARB medications in females over 65 were protective factors against in-hospital mortality of COVID-19 patients.

## 7. Declarations

### 7.1. Acknowledgments

The authors express their appreciation to the participants and the personnel of Imam Hossien hospital for their collaboration. We acknowledge the team of professional nurses of Shahid Beheshti University of Medical Science for their effort in data collection (Mrs. Golnoosh Mortezaee, Effat Taheri, Ghodsi Najari, Faezeh Nesaei, Faezeh Fakour, Ghazaleh Amanabadi, and Vida Torabi) and Maedeh Sayad for dedication to data entry. 

### 7.2. Funding

This work was supported by the Prevention of Cardiovascular Disease Research Center, Shahid Beheshti University of Medical Science, Tehran, Iran. 

### 7.3. Conflict of interest statement

The authors have declared that no competing interests exist.

### 7.4. Authors' contributions

All authors met the four criteria for authorship contribution based on the recommendations of the international committee of medical journal editors.
